# Supplementary figures and images for: Distinct cellular and molecular mechanisms contribute to the specificity of the two Drosophila melanogaster chitin synthases in chitin deposition
Source: PLoS Genet. 2025 Sep 8;21(9):e1011847. doi: 10.1371/journal.pgen.1011847 (PMC12431666; doi:10.1371/journal.pgen.1011847)

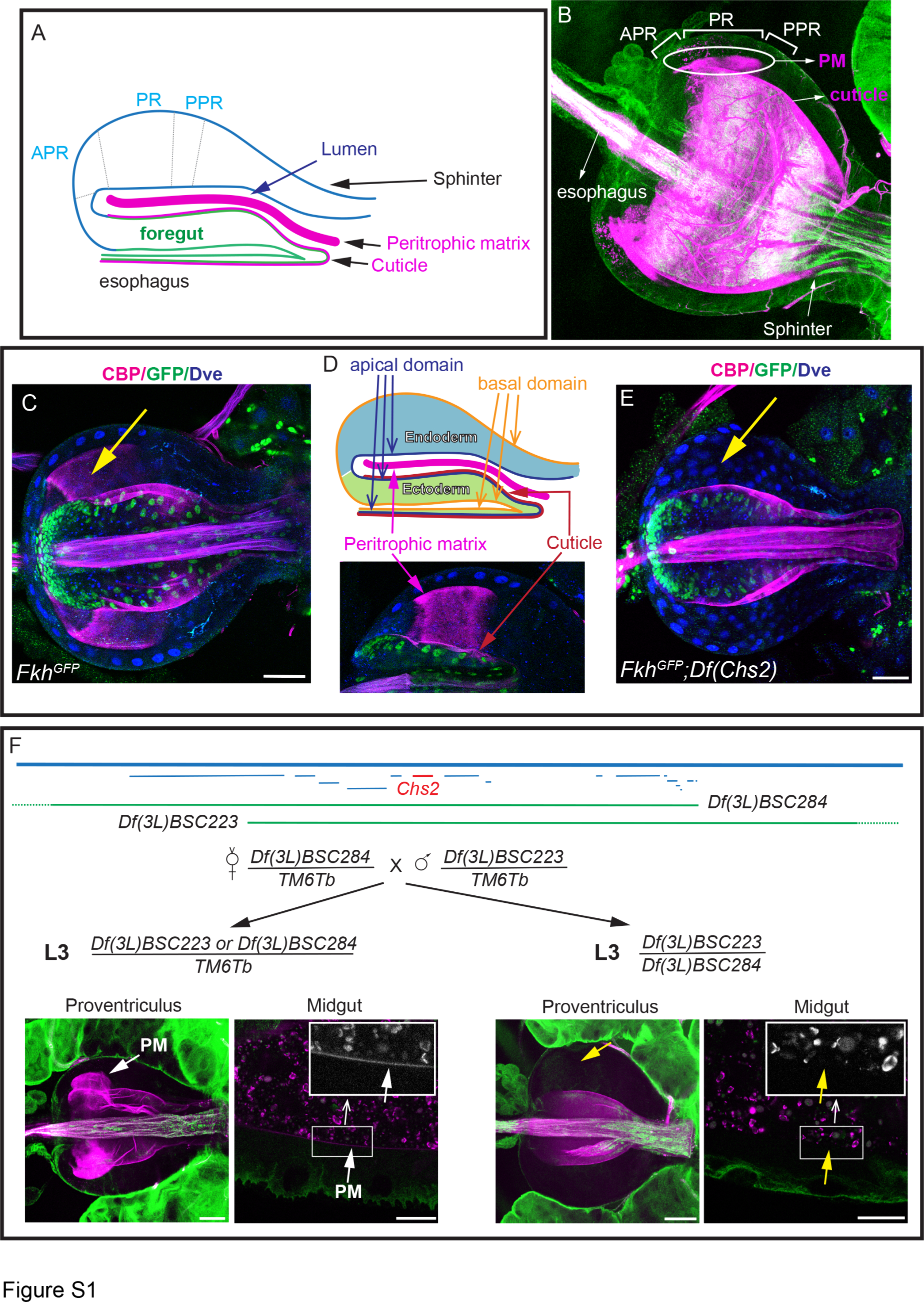

Supplement: S1 Fig — (A) Schematics of a sagittal section of half proventriculus indicating the position of the APR,PR and PPR midgut cells. (B) Confocal projection of a wild type proventriculus of a L3 larva stained for chitin (CBP, magenta) and α-Spec as cellular marker (green). PR, APR and PPR cells are indicated. Note the enrichment of chitin in the region of PR cells, corresponding to the PM. (C-E) Chitin deposition in relation to ectodermal/endodermal regions. The Fkh pattern (green) marks the ectodermal cells, which deposit a cuticle along their apical domain (magenta) (C,D). Dve marks the endodermal cells (blue), which deposit a PM along their apical domain (yellow arrow in C,D). In conditions of Chs2 absence, no PM is detected in the endodermal regions (yellow arrow in E), but ectodermal cells still deposit a cuticle (E). Scheme in D indicates the apical and basal domains in the different regions in the proventriculus. (C) Schematics representing the chromosomal region of Chs2 and the deficiencies used to remove the gene. The combination of the deficiencies gives rise to L3 scapers that lack chitin enrichment in the proventriculus and lining the midgut (yellow arrows and magnification of boxed region in grey), compared to their sibling heterozygote larvae (white arrows point to PM, magnification of boxed region in grey). Scale bars C,E,F-proventriculus 50 μm; F-midgut 20 μm. (TIF) [file pgen.1011847.s001.tif]

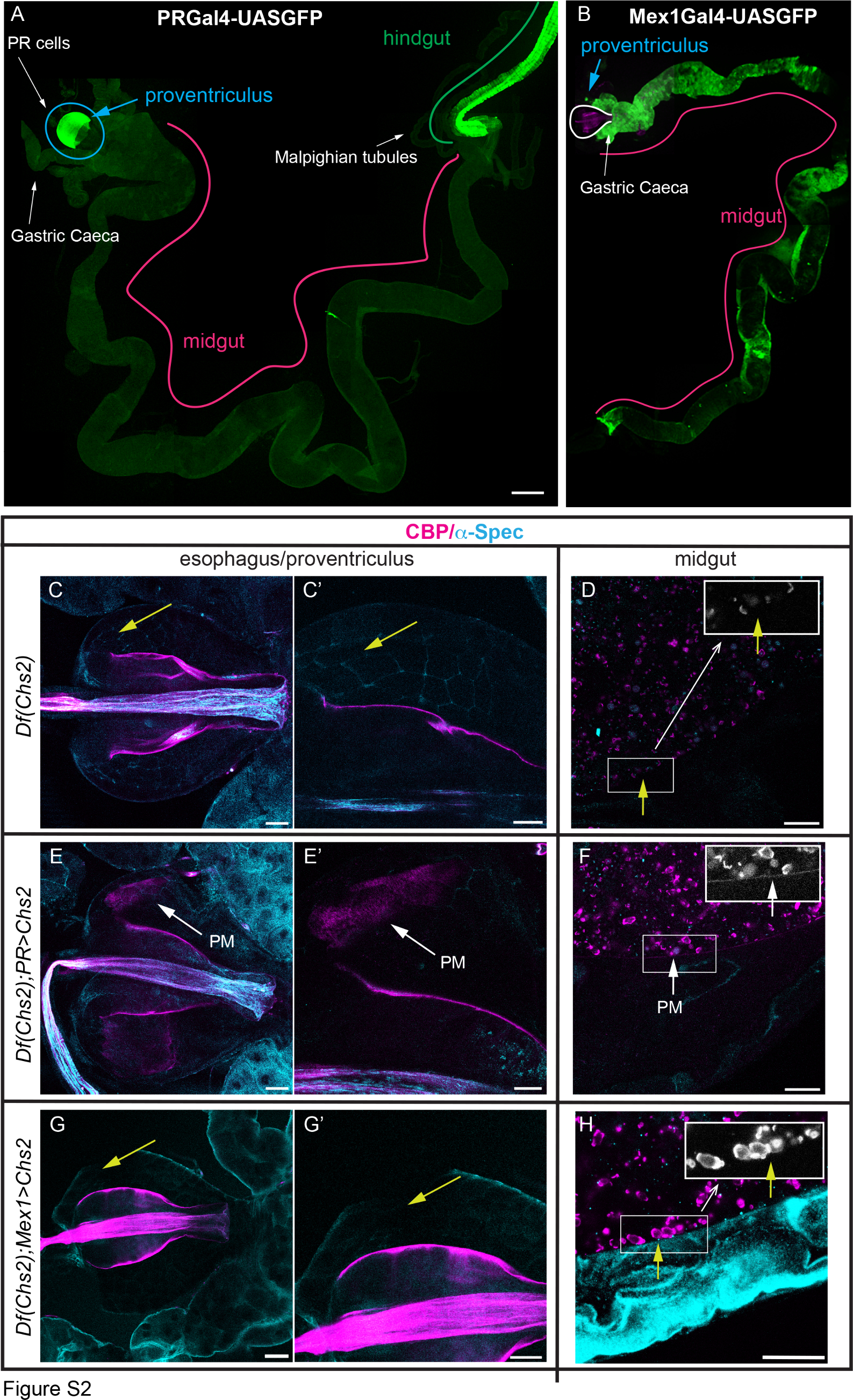

Supplement: S2 Fig — (A,B) Montage of several confocal projections stained for GFP (green) to include the whole digestive tract. Note that the PRGal4 line used is expressed only in the PR cells and in the hindgut. Mex1 is expressed in the midgut and is absent in the proventriculus. (C-H) L3 digestive tracts stained for chitin using CBP (magenta) and α-Spec in the indicated genotypes. All images correspond to single confocal sections. No chitin in the PM in L3 Df(Chs2) escapers is observed (yellow arrows in C,D). Expression of Chs2 in PR cells rescues chitin deposition in the PM (white arrows in E,F). Expression of Chs2 in the midgut does not rescue chitin deposition in the PR region or along the midgut (yellow arrows in G,H). Scale bars A 200 μm; C,E,G 50 μm; C’,E’,G’,D,F,H 20 μm. (TIF) [file pgen.1011847.s002.tif]

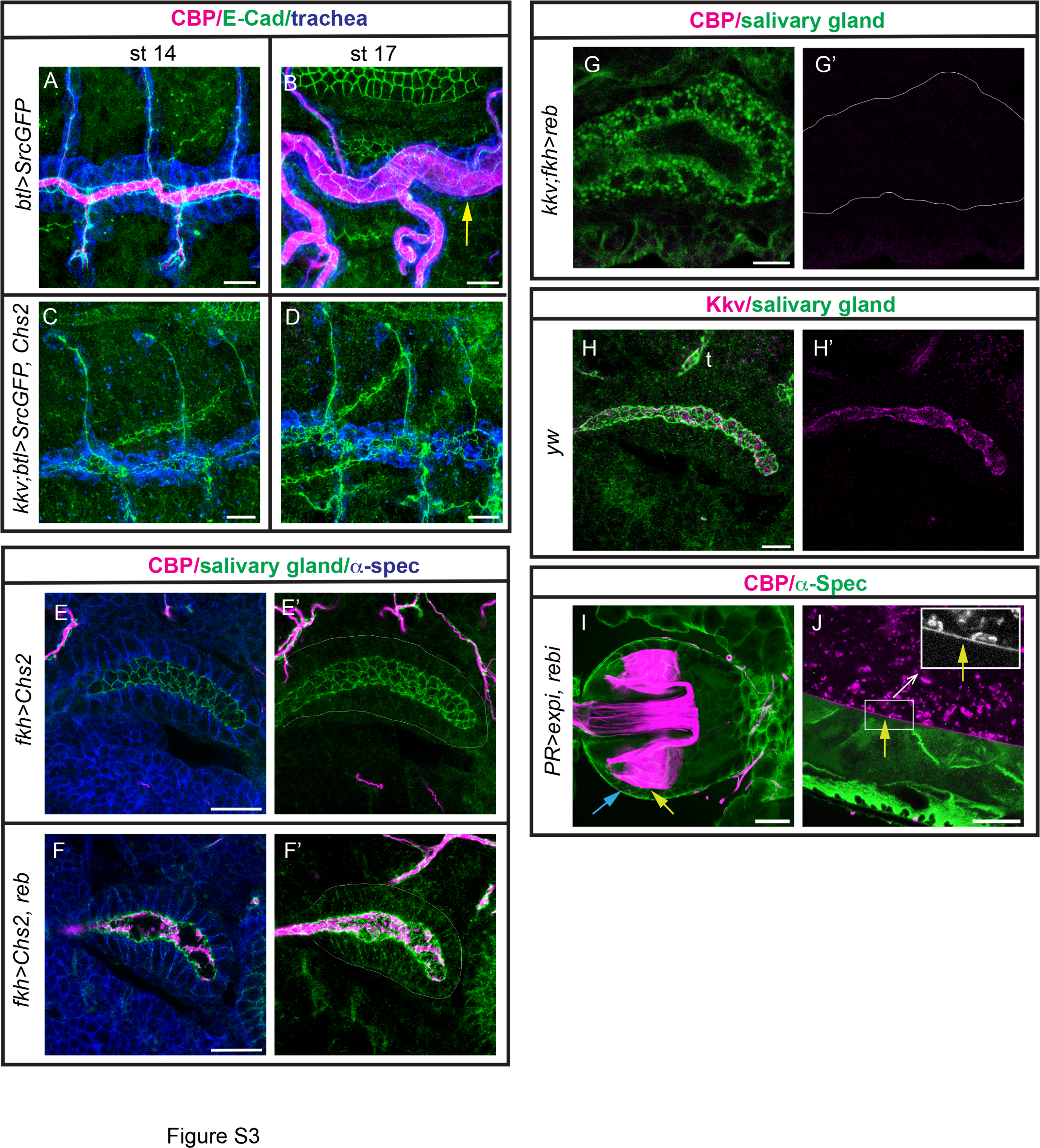

Supplement: S3 Fig — (A-D) Confocal projections showing dorso-lateral views of the trachea stained for chitin (CBP, magenta), GFP to visualise the tracheal cells (blue) and E-Cad (green). A luminal filament assembles at early stages (A) and a cuticle with the taenidial pattern forms later (B) in the wild type. Chitin deposition is not rescued in kkv mutants when adding back Chs2 (C,D). (E-H) Single sections (E-G) or projections (E’-G’, H,H’) of salivary glands stained for chitin or Kkv (magenta) and to highlight the salivary glands (green) in the indicated genotypes. Chs2 cannot deposit chitin intracellularly when expressed alone (E). In combination with Reb (F) there is some deposition of chitin in the lumen (as when expressing Reb alone, Fig 3N). In kkv mutants, the expression of Reb cannot promote the deposition of chitin in salivary glands (G). This indicates that the luminal staining in fkh > reb conditions is due to the presence of Kkv. Accordingly, Kkv protein is detected in the salivary glands (H). (I) Single sections of proventriculus and midgut stained for chitin (CBP, magenta) and α-Spec as cellular markers (green). The concomitant downregulation of exp and reb in PR cells does not prevent chitin deposition in the proventriculus (yellow arrow in I) and midgut (yellow arrow in J, magnification of boxed region in grey). Chitin is also detected in intracellular punctae in PR cells (blue arrow in I). Scale bars A-H 10 μm; I 50μm;J 20 μm. (TIF) [file pgen.1011847.s003.tif]

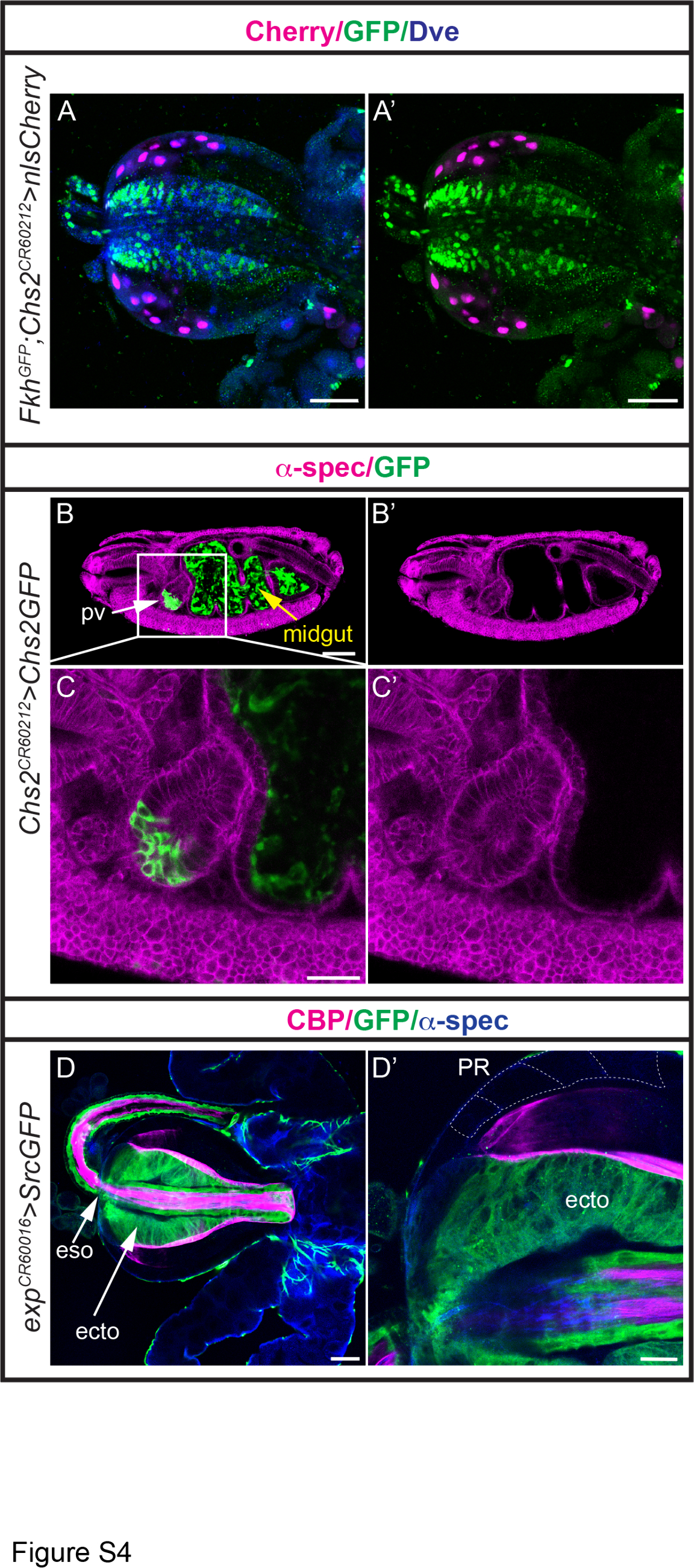

Supplement: S4 Fig — Expression pattern of Chs2 in the embryo and proventriculus and exp in the proventriculus. (A) Confocal projection of FkhGFP; Chs2CR60212-TG4.0-UASnlsCherry proventriculus stained for GFP (green, to visualise Fkh), Cherry (magenta, to visualise Chs2 pattern) and Dve (blue). Chs2 colocalises with Dve and is absent from the ectodermal region. (A,B) Single sections of embryos at stage 16 stained with α-Spec (magenta) to visualise the cells and GFP to visualise Chs2 pattern. Note the expression in a few cells in the proventriculus. B corresponds to a magnification of A. (C) Single sections of proventriculus stained for chitin (magenta), GFP (green) to visualise Exp expression and α-Spec (blue) to visualise the cells. Exp is expressed in the ectodermal region of the proventriculus and the esophagus and it is absent in PR cells. ecto-ectoderm; eso-esophagus Scale bars A,B,D 50 μm; C,D’ 20 μm (TIF) [file pgen.1011847.s004.tif]

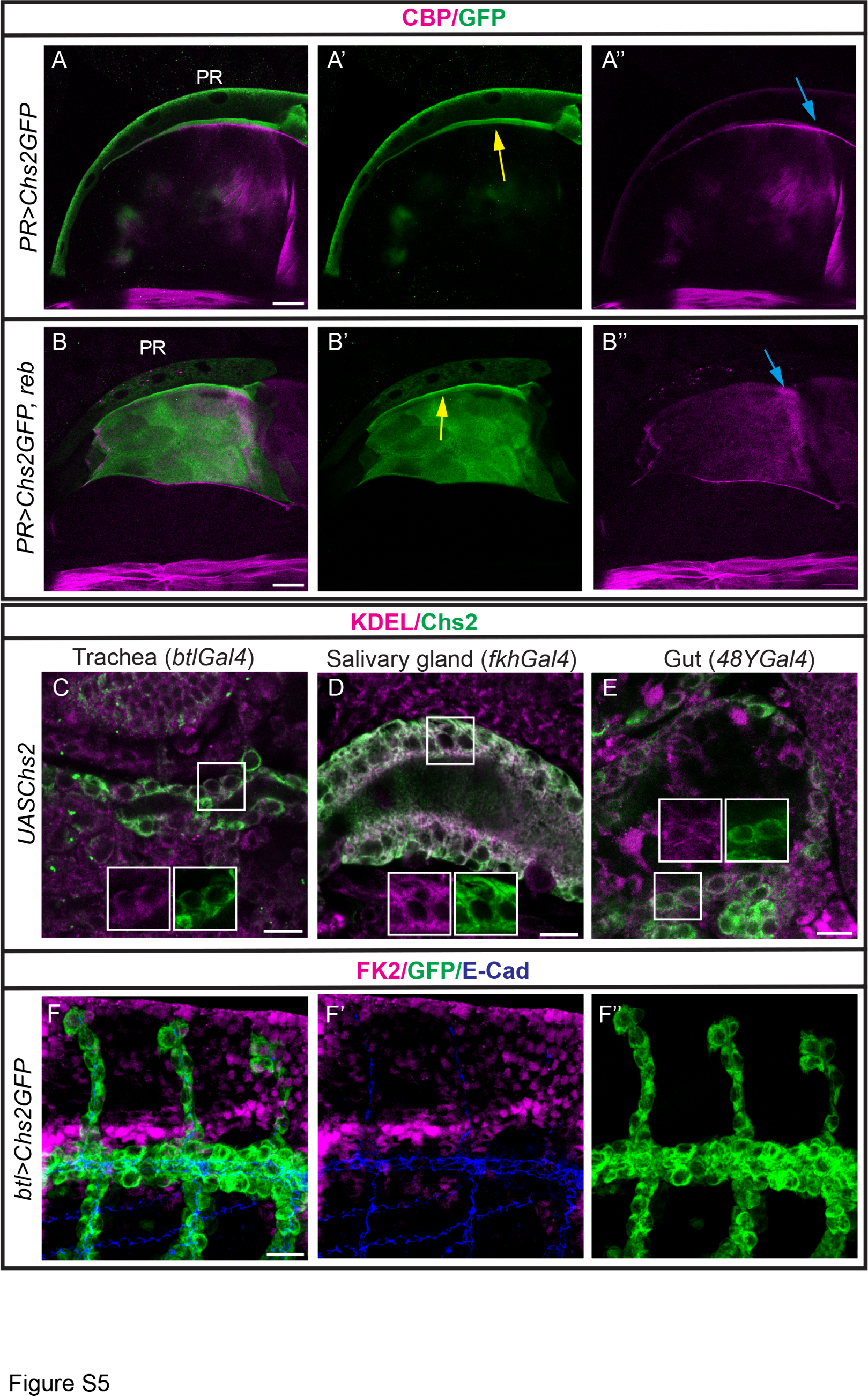

Supplement: S5 Fig — (A,B) Single sections of proventriculus stained for chitin (magenta) and GFP (green) to visualise Chs2 subcellular localisation in PR cells in the indicated genotypes. Chs2 is enriched at the apical domain of PR cells (yellow arrows in A’, B’) in the absence or presence of Reb, and its accumulation correlates with a clear enrichment of chitin. Note the fibrous aspect of chitin in the PM (blue arrows in A’‘, B’‘). (C-E) Single sections of embryos stained for KDEL (magenta), anti-Chs2 to visualise Chs2 in the indicated genotypes. Chs2 localises intracellularly, largely colocalising with the ER marker in all embryonic tissues analysed. (F) Single section stained for Fk2 (magenta), GFP to visualise Chs2 (green), and E-Cad to visualise the cells (blue). Chs2GFP does not colocalise with FK2. Scale bars A, B 20 μm, C-F 10 μm. (TIF) [file pgen.1011847.s005.tif]

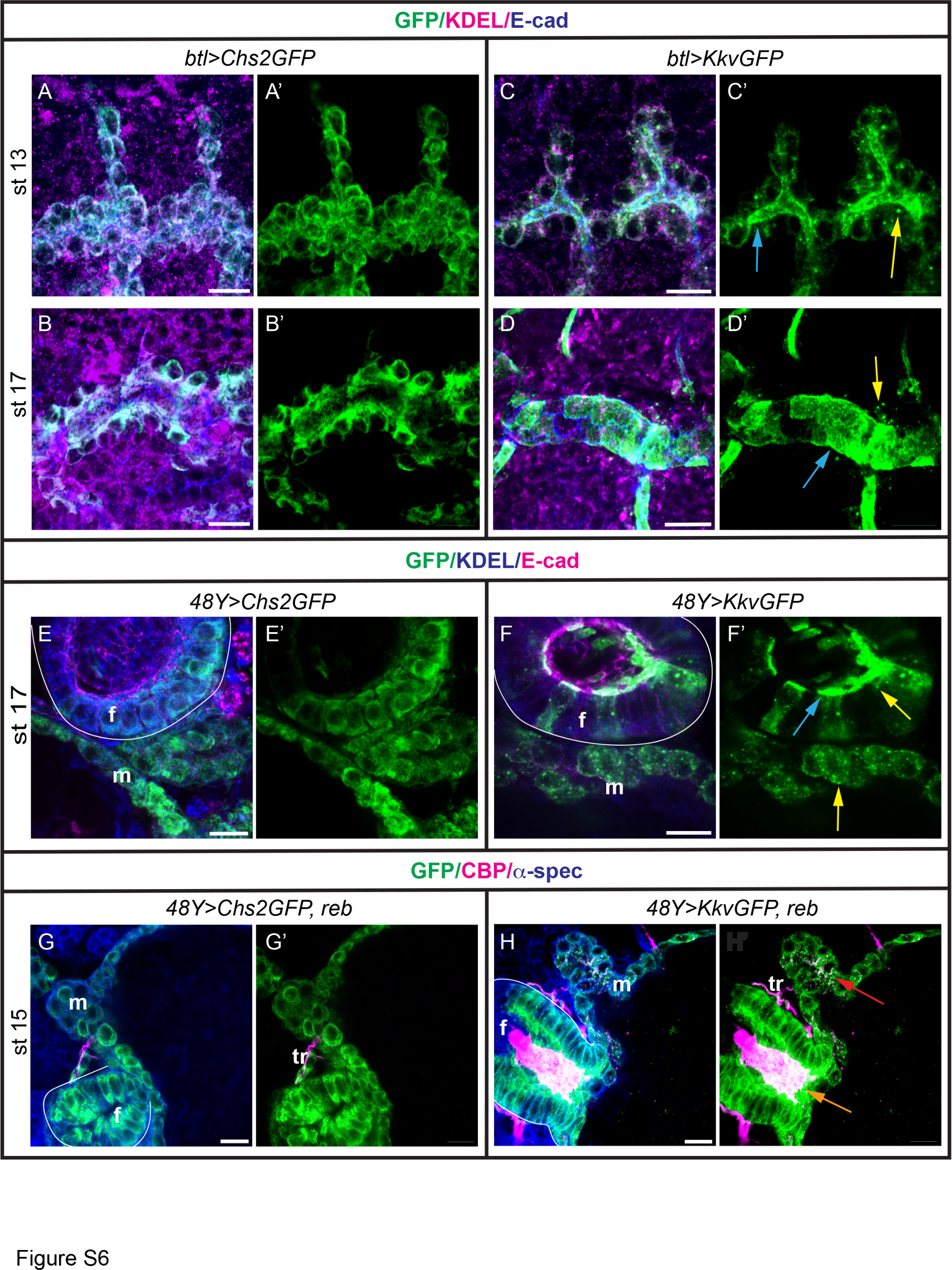

Supplement: S6 Fig — Confocal projections of embryonic trachea or intestinal tracts (at the indicated stages) upon Chs2GFP or KkvGFP overexpression using btlgal4 (expressed in tracheal cells) or 48YGal4 (expressed in the ectodermal-foregut and endodermal-midgut). Embryos are stained for the indicated antibodies. In the trachea, KkvGFP can be found in the whole cell, particularly at early stages (C), but it is predominantly localised apically (blue arrows in C,D) and detected in intracellular vesicles (yellow arrows C,D). In contrast, Chs2GFP localises in the whole cell (A,B). In the intestinal tract, KkvGFP localises apically in the foregut region (f, encircled in white, blue arrow in F’) and it is diffusely localised within the cell in the midgut (m). In both regions, KkvGFP is detected in vesicles (yellow arrows in F). The pattern correlates with the ability of Kkv to deposit chitin extracellulaly in the foregut (encircled in white, orange arrow in H’) or intracellularly in the midgut (red arrow in H’). In contrast, Chs2 localises diffusely in the whole cell in the foregut (encircled in white in E, G) or midgut regions. tr, trachea; f, foregut; m, midgut. Scale bars 10 μm. (TIF) [file pgen.1011847.s006.tif]

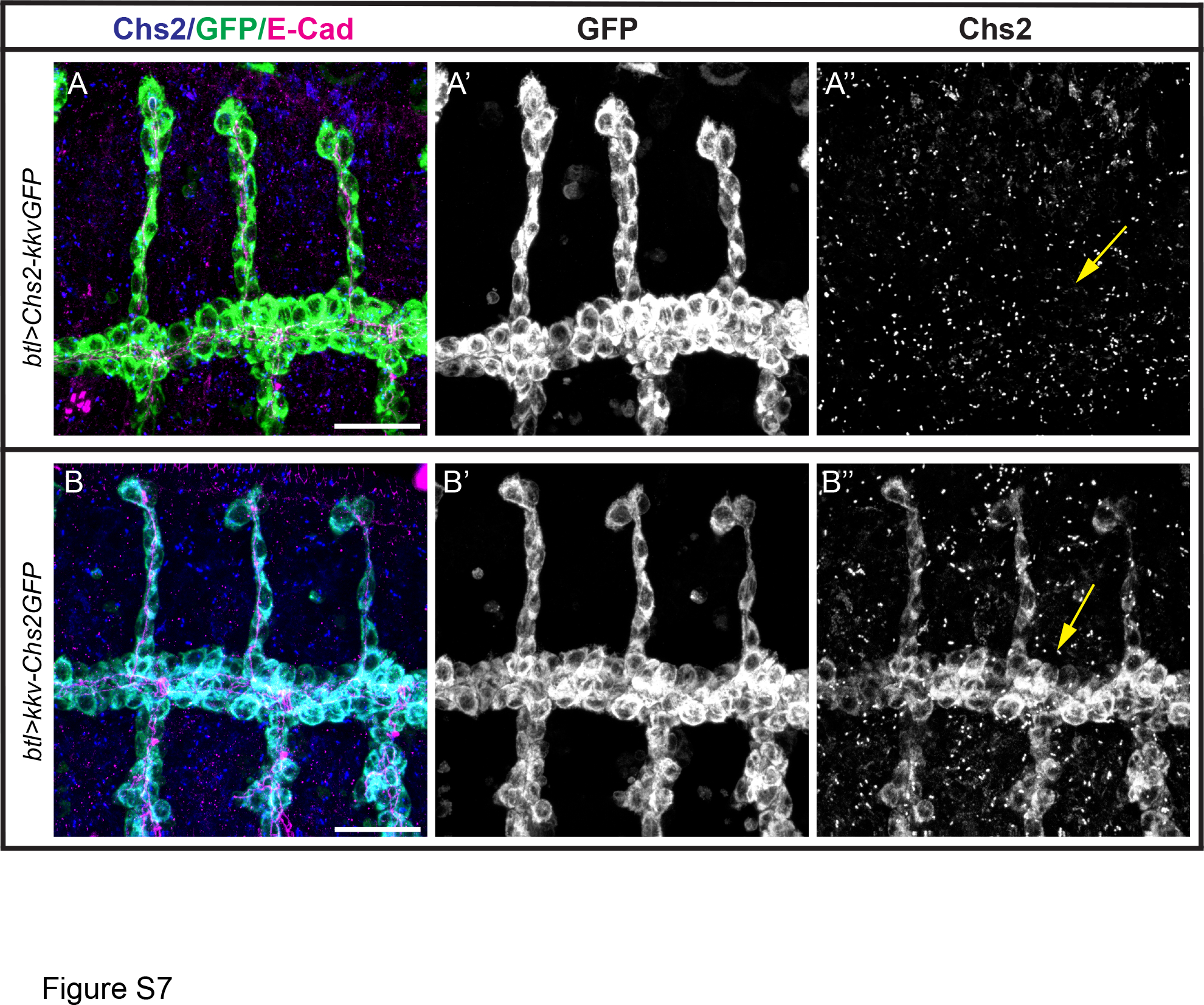

Supplement: S7 Fig — (A,B) Confocal projections showing dorso-lateral views of the trachea stained for GFP (green), Chs2 antibody generated in the lab (blue, see Materials and methods) and E-Cad (magenta) to follow the tubes in the indicated genotypes. Both chimeras are tagged with GFP (A’, B’). Only kkv-Chs2GFP is recognised by Chs2 antibody (yellow arrow in B’‘), which was generated against a region in the Carboxi-terminal domain of Chs2 that is not present in Chs2-kkvGFP (yellow arrow in A’‘). Scale bars 20 mm. (TIF) [file pgen.1011847.s007.tif]
