# Supplementary material for: Distinct cellular and molecular mechanisms contribute to the specificity of the two Drosophila melanogaster chitin synthases in chitin deposition
Source: PLoS Genet. 2025 Sep 8;21(9):e1011847. doi: 10.1371/journal.pgen.1011847 (PMC12431666; doi:10.1371/journal.pgen.1011847)
Supplement: Attachment — Submitted filename: 154233_1_rebuttal_3376362_svvy9d.pdf [file pgen.1011847.s010.pdf]

Full Revision

Review COMMONS

Manuscript number: RC-2025- 02861
Corresponding author(s): Marta Llimargas

[Please use this template only if the submitted manuscript should be considered by the affiliate journal as a full revision in response to the points raised by the reviewers.

If you wish to submit a preliminary revision with a revision plan, please use our "Revision Plan" template. ***It is important to use the appropriate template to clearly inform the editors of your intentions.****]*

1. General Statements [optional]

This section is optional. Insert here any general statements you wish to make about the goal of the study or about the reviews.

We have thoroughly addressed all concerns raised by the reviewers, either by conducting new experiments or by clarifying the text. We believe that the additions and revisions have significantly strengthened the manuscript and enhanced its potential to represent a step-forward in the field. Below, we are providing a detailed point-by-point response to the reviewers' concerns. The changes made to the manuscript are highlighted in yellow in the revised version.

2. Point-by-point description of the revisions

This section is mandatory. Please insert a point-by-point reply describing the revisions that were already carried out and included in the transferred manuscript.

Reviewer #1 (Evidence, reproducibility and clarity (Required)):

Summary:
Chitin is a critical component of the extracellular matrix of arthropods and plays an essential role in the development and protection of insects. There are two chitin synthases in insects: Type A (exoskeletons) and Type B (for the peritrophic matrix in the gut). The study aims to investigate the specificity and mechanisms of the two chitin synthases in D. melanogaster and to clarify whether they are functionally interchangeable. Various genetic manipulations and fluorescence-based labeling were used to analyze the expression, localization, and function of Kkv and Chs2 in different tissues.
Chs2 is expressed in the PR cells of the proventriculus and is required for chitin deposition in the peritrophic matrix. Kkv can deposit chitin in ectodermal tissues but not in the peritrophic matrix, whereas Chs2 can deposit chitin in the peritrophic matrix but not in ectodermal tissues. The subcellular localization of chitin synthases is specific to the tissues in which they are expressed. Kkv localizes apically in ectodermal tissues, whereas Chs2 localizes apically in the PR cells of the proventriculus. Altogether, Kkv and Chs2 cannot replace each other. The

specificity of chitin synthases in D. melanogaster relies on distinct cellular and molecular mechanisms, including intracellular transport pathways and the specific molecular machinery for chitin deposition.

Congratulations on this incredible story and manuscript, which is straightforward and well-written. However, I have some comments that may help to improve it.

We thank the reviewer for this very positive comment. We have addressed all comments to clarify and improve our manuscript.

Major comments:
1.) Funny thing: the Chs2 mutant larva shows a magenta staining below the chitin accumulation of the esophagus, which looks like a question mark in 1H but cannot be found in control. Is that trachea reaching the pv?

We assume that the reviewer refers to Fig 1N. As the reviewer suspects, this corresponds to a piece of trachea. Figure 1N shows a single section, making it difficult to identify what this staining corresponds to. We are providing below a projection of several sections where it is easier to identify the staining as tracheal tissue (arrow).

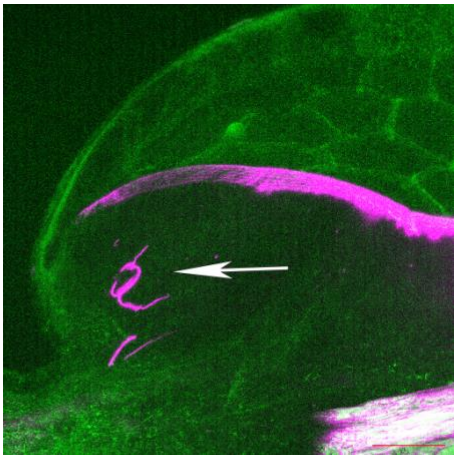

We are now marking this pattern as trachea (tr) in the manuscript Figure 1N

2.) Also, though it is evident that the PM chitin is lost in Ch2 mutants, could it be that the region is disturbed and cells express somewhere else chitin? There are papers by Fuß and Hoch (e.g., Mech of Dev, 79, 1998; Josten, Fuß et al., Dev. Biol.267, 2004) using markers such as Dve, Fkh, Wg, Delta, and Notch, etc. for precisely marking the endodermal/ectodermal region in the embryonic foregut/proventriculus. It would be beneficial to show, along with chitin and Chs expression patterns, the ectoderm/endoderm cells. This is particularly important as the authors report endodermal expression of Chs2 in embryos but don't use co-markers of the endodermal cells.

We agree with the reviewer that this is an important issue and we note that Reviewer 2 also raised the same point. Therefore, we have addressed this issue.

We obtained an antibody against Dve, kindly provided by Dr. Hideki Nakagoshi. Dve marks the endodermal region in the proventriculus (Fuss and Hoch, 1998, Fuss et al., 2004, Nakagoshi et al., 1998).This antibody worked nicely in our dissected L3 digestive tracts and allowed us to mark the endodermal region. We also obtained an antibody against Fkh, kindly provided by Dr. Pilar Carrera. Fkh marks the ectodermal foregut cells (Fuss and Hoch, 1998, Fuss et al., 2004). While, in our hands, this antibody performed well in embryonic tissues, we observed no staining in our dissected L3 digestive tracts. The reason for this is unclear, but we suspect technical limitations may be responsible (the ectodermal region of the proventriculus is very internal, potentially hindering antibody penetration). To circumvent this inconvenience, we tested a Fkh^{GFP} tagged allele available in Bloomington Stock Center. Fortunately, we were able to detect GFP in ectodermal cells of L3 carrying this allele. Using this approach, we conducted experiments to detect Fkh and Dve in the wild type or in Df(Chs2) conditions (Fig S1). In addition, we used these markers to map the expression of Kkv and Chs2 in the proventriculus (Fig 4).

Altogether the results using these endodermal/ectodermal markers confirmed the presence of a cuticle adjacent to the FkhGFP-positive cells and a PM adjacent to the PR cells, marked by Dve. This PM is absent in Df(Chs2) L3 escapers, however, the general pattern of Fkh/Dve expression is not affected. Finally, we show that Chs2-expressing cells are positive for Dve while Kkv-expressing cells are not. We were unable to conduct an experiment demonstrating Kkv and Fkh co-expression due to technical incompatibilities, as both genes require the use of GFP-tagged alleles to visualise their expression. However, we believe that our imaging of Dve/Kkv clearly shows that Kkv expressing cells lack Dve expression and are localised in the internal (ectodermal) region of the proventriculus (Fig 4E).

3.) The origin of midgut chitin accumulation is unclear. Chitin can come from yeast paster. Can the authors check kkv and chs2 mutants for food passage and test starving L1 larvae to detect chitin accumulation in the midgut without feeding them?

This is a very interesting point that has also intrigued us.

We observed that, in addition to the PM layer lining the midgut epithelium, CBP staining also revealed a distinct luminal pattern. Our initial hypothesis was that this pattern corresponded to the PM. However, its presence in Df(Chs2) larval escapers clearly indicates that this is not the case. Unfortunately, we cannot assess this pattern in *kkv* mutants, as these die at eclosion and do not proceed to larva stages.

As the reviewer suggests, a likely possibility is that the luminal pattern originates from components in the food. These could correspond to yeast, as suggested by the reviewer, or possibly remnants of dead larvae present in the media (although Drosophila is considered herbivore in absence of nutritional stress).

To assess whether the luminal pattern originates from the food we conducted two independent experiments. In **experiment 1**, we collected larvae reared under normal food conditions. Newly emerged L3 larvae were transferred in small numbers to minimise cannibalism (Ahmad et al., 2015) to new Petri plates containing moist paper. Larvae were starved for 3,4 or 5 days. Larvae starved for more than 5 days did not survive. We then dissected the guts and analysed CBP staining. We observed the presence of luminal CBP staining in these larvae, along with the typical PM signal in the proventriculus and along the midgut. In **experiment 2**, we collected larvae directly on agar plates containing only agar (without yeast or any other nutrients). We allowed the larvae

to develop. These larvae showed minimal growth. We dissected the guts of these small larvae (which were challenging to dissect) and analysed CBP staining. Again, we detected presence of luminal CBP staining.

These experiments indicate that, despite starvation, a luminal chitin pattern is still detected, suggesting that it is unlikely to originate from food. However, we cannot unequivocally rule out the possibility that the cannibalistic, detrivorous or carnivorous behavior of the nutrionally stressed larvae (Ahmad et al., 2015) in our experiments may influence the results. Therefore, more experiments would be required to address this point.

In summary, while we cannot provide a definitive answer to the reviewer's question, nor fully satisfy our own curiosity, we would like to note that this specific observation is unrelated to the main focus of our study, as we have confirmed that the luminal pattern is not dependent on Chs2 function.

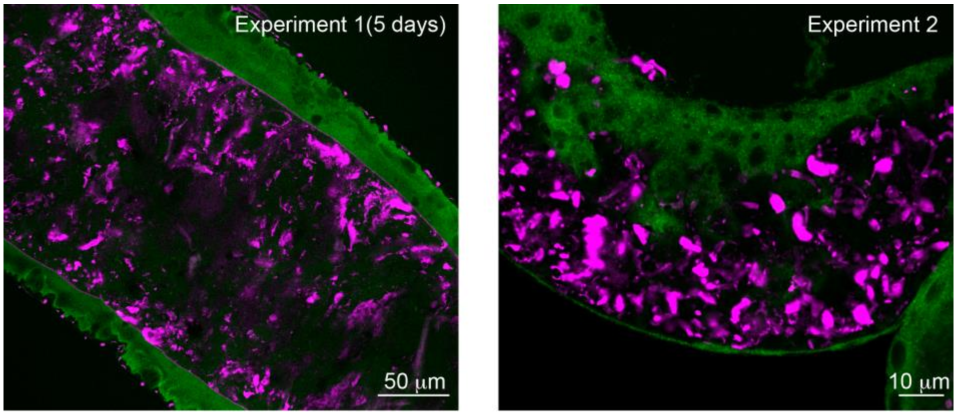

Portions of midgut of starved larvae under the regimes indicated, stained for chitin (CBP, magenta). Note the presence of the luminal chitin pattern in the midgut

4.) Subcellular localization assays require improved analysis, such as a co-marker for the apical membrane and statistical analysis with co-localization tools, showing the overlap at the membrane and intracellularly with membrane co-markers and KDEL.

We have addressed the point raised by the reviewer. To analyse and quantify Chs2 subcellular localisation, particularly considering the observed pattern, we decided to use both a membrane and an ER marker. As a membrane marker we used srcGFP expressed in tracheal cells (see answer to point 7 of Reviewer 1) and as an ER marker we used KDEL. In this analysis, tracheal cells also expressed Chs2, which was visualised using the Chs2 antibody generated in the lab.

To assess the colocalisation of Chs2 with each marker we used the JaCop pluggin in Fiji. We analysed individual cells from different embryos stained for membrane/ER/Chs2 using single confocal sections (to avoid artificial colocalisation). Images were processed as described in Materials and Methods. We obtained the Pearson's correlation coefficient (r), which measures the degree of colocalisation, for Chs2/srcGFP and Chs2/KDEL, n=36 cells from 9 different embryos. The average r value for Chs2/srcGFP was 0,064, while the average for Chs2/KDEL was around 0,7. r ranges between -1 and 1, where 1 indicates perfect correlation, 0 no correlation, and -1 perfect anti-correlation. Typically, an r value of 0.7 and above is considered a strong positive

correlation, whereas a value below 0,1 is regarded as very weak or no correlation. Thus, our colocalisation analysis supports the hypothesis that Chs2 is primarily retained in the ER when expressed in non-endogenous tissues, likely unable to reach the membrane.

We have reorganised the figures and now present an example of Chs2/srcGFP/KDEL subcellular localisation in tracheal cells and the colocalisation analysis in Fig 5H. The colocalisation analysis is described in the Materials and Methods section.

Minor comments:

5.) The authors used "L3 larval escapers." It would be interesting to know if the lack of Chs2 and the peritrophic matrix cause any physiological defects or lethality.

The point raised by the reviewer is very interesting and relevant. The peritrophic matrix is proposed to play several important physiological roles, including the spatial organisation of the digestive process, increasing digestive efficiency, protection against toxins and pathogens, and serving as a mechanical barrier. Therefore, it is expected that the absence of chitin in the PM of the Df(Chs2) larval escapers may cause various physiological effects.

Analysing these effects is a complex task, and it constitutes an entire research project on its own. In addressing the physiological requirements of the PM, we aim to analyse adult flies and assess various parameters, including viability, digestive transit dynamics, gut integrity, resistance to infections, fitness and fertility.

A critical initial challenge in conducting a comprehensive analysis of the physiological requirements of the PM is identifying a suitable condition to evaluate the absence of Chs2. In this work we are using a combination of two overlapping deficiencies that uncover Chs2, along with a few additional genes (as indicated in Fig S1F). This deficiency condition presents two major inconveniences: first, the observed defects could be caused or influenced by the absence of genes other than Chs2, preventing us from conclusively attributing the defects to Chs2 loss (unless we rescued the defects by adding Chs2 back as we did in the manuscript). Second, the larva escapers, which are rare, do not survive to adulthood (indicating lethality but preventing us from analysing specific physiological aspects).

To overcome these limitations, we are currently working to identify a genetic condition in which we can specifically analyse the absence of Chs2. We have identified several available RNAi lines and we are testing their efficiency in preventing chitin deposition in the PM. Additionally, we are characterising a putative null Chs2 allele, $Chs2^{CR60212\text{-}TG4.0}$. This stock contains a Trojan-GAL4 gene trap sequence in the third intron, inserted via CRISPR/Cas9. As described in Flybase (https://flybase.org/), the inserted cassette contains a 'Trojan GAL4' gene trap element composed of a splice acceptor site followed by the T2A peptide, the GAL4 coding sequence and an SV40 polyadenylation signal. When inserted in a coding intron in the correct orientation, the cassette should result in truncation of the trapped gene product and expression of GAL4 under the control of the regulatory sequences of the trapped gene. We already know that, when crossed to a reporter line (e.g. UAS-GFP or UAS-nlsCherry) this line reproduces the Chs2 expression pattern, suggesting that the insertion may generate a truncated Chs2 protein. This line would represent an ideal tool to assess the absence of Chs2, and we are currently characterising it for further analysis

In summary, we fully agree with the reviewer that investigating the physiological requirements of the PM is a compelling area of research, and we are actively addressing this question. However, this investigation constitutes a substantial and independent research effort that we believe is beyond the scope of the current manuscript at this stage.

6.) The order identifiers are missing for materials and antibodies, e.g., anti-GFP (Abcam), but Abcam provides several ant-GFP; which was used? Please provide order numbers that guarantee the repeatability for others.

We have now added all identifiers for materials and reagents used, in the materials and methods section.

7.) Figure S5C, C', what marks GFP (blue) in the trachea? Maybe I have overlooked the description. What is UASsrcGFP? What is the origin of this line?

We apologise for not providing a more detailed description of the UASsrcGFP line. This line corresponds to RRID BDSC#5432, as now indicated in Materials and Methods section.
In this transgene, the UAS regulatory sequences drive the expression of GFP fused to Tag:Myr(v-src). As described in Flybase (https://flybase.org/), the P(UAS-srcEGFP) construct contains the 14 aa myristylation domain of v-src fused to EGFP. This tag is commonly used to target proteins of interest to the plasma membrane. The construct was generated by Eric Spana and is available in Drosophila stock centers.

We typically use this transgene as a plasma membrane marker to outline cell membrane contours. In our experiments, srcGFP, under the control of the btlGal4 promoter, was used to visualise the membrane of tracheal cells in relation to Chs2 accumulation. As indicated in point 4, we have now transferred the images of srcGFP/Chs2/KDEL to the main Figures and used it for colocalisation analyses.

8.) The authors claim that they validated the anti-Chs2 antibody. However, they show only that it recognizes a Cht2 epitope via ectopic expression. For more profound validation, immune staining is required in deletion mutants, upon knockdown, or upon expression of recombinant proteins, which is not shown.

We generated an antibody against Chs2. We found that the antibody does not reliably detect the endogenous Chs2 protein, and so we find no pattern in the proventriculus or any other tissue in our immunostainings. It is very possible that the combination of low endogenous levels of Chs2 with a sub-optimal antibody (or low titer) leads to this result. In any case, as the antibody does not detect endogenous Chs2, it cannot be validated by analysing the expression upon Chs2 knockdown. In contrast, our antibody clearly detects specific staining in various tissues (e.g. trachea, salivary glands, gut) when Chs2 is expressed using the Gal4/UAS system, confirming its specificity for Chs2. It is worth to point that it is not unusual to find antibodies that are not sensitive enough to detect endogenous proteins but can detect overexpressed proteins (e.g (Lebreton and Casanova, 2016)).

As an additional way to validate the specificity of our antibody, we have used the chimeras generated, as suggested by the reviewer. As indicated in the Materials and Methods section, the Anti-Chs2 was generated against a region comprising 1222-1383 aa in Chs2, with low homology to Kkv. This region is present in the kkv-Chs2GFP chimera but absent in Chs2-KkvGFP (see Fig

7A). Accordingly, our antibody recognises kkv-Chs2GFP but does not recognise Chs2-KkvGFP (Fig S7).

We have revised the text in chapter 6 (6. Subcellular localisation of Chs2 in endogenous and ectopic tissues) to clarify these points and we have added the validation of the antibody using the chimeras in chapter 8 (8. Analysis of Chs2-Kkv chimeras) and Fig S7

9) The legend and text explaining Fig. 4 D-E' can be improved. The authors used the Crimic line, which is integrated into the third ("coding") intron. This orientation can lead to the expression of Gal4 and cause a truncated version of the protein (according to Flybase). Is Chs2 expression reduced in the crimic mutant? If the mutation causes expression of a truncated version, the Chs2 antibody may not be able to detect it as it recognizes a fragment between 1222 and 1383 aa?
Also, I'm unsure whether the Chs2 antibody or GFP was used to detect expression in PR cells. The authors describe using Ch2CR60212>SrcGFP together with Chs2+ specific antibodies.

We apologise for the confusion.
As the reviewer points, $Chs2^{CR60212\text{-}TG4.0}$ contains a Trojan-GAL4 gene trap sequence in the third intron, inserted via CRISPR/Cas9. As described in Flybase (https://flybase.org/), the inserted cassette contains a 'Trojan GAL4' gene trap element composed of a splice acceptor site followed by the T2A peptide, the GAL4 coding sequence and an SV40 polyadenylation signal. When inserted in a coding intron in the correct orientation, the cassette should result in truncation of the trapped gene product and expression of GAL4 under the control of the regulatory sequences of the trapped gene.

We found that when crossed to UAS-GFP or UAS-nlsCherry, this line reproduces a expression pattern that must correspond to Chs2. As the antibody that we generated is not suitable for detecting Chs2 endogenous expression, we resorted to using this combination, $Chs2^{CR60212\text{-}TG4.0}$ crossed to a reporter line (such asUAS-GFP or UAS-nlsCherry), to visualise Chs2 expression by staining for GFP/Cherry in the intestinal tract and in the embryo (Figures 4 and S4).

We realise that the Figure labelling we used in our original submission is very misleading, and we apologise for this. In the original figures we had labelled the staining combination with Kkv, Chs2, Exp as if we had used these antibodies. However, in all cases, we used GFP to visualise the pattern of these proteins in the genetic combinations indicated in the figures. We have corrected this in our revised version. We have also updated the text (Chapter 5), figures and figure legends.

As the reviewer points, the insertion in $Chs2^{CR60212\text{-}TG4.0}$ is likely to generate a truncated Chs2 protein. We cannot confirm this using the Chs2 antibody we generated because it does not recognise the endogenous Chs2 pattern. Nevertheless, as indicated in point 5, we are currently characterising this line. Our preliminary results indicate a high complexity of effects from this allele that require thorough analysis, as it may be acting as a dominant negative.

Reviewer #1 (Significance (Required)):

Significance:
The manuscript's strength and most important aspects are the genetic analysis, expression, and localization studies of the two Chitin synthases in Drosophila embryos and larvae. However, beyond this manuscript, the development of mechanistic details, such as interaction partners

that trigger secretion and action at the apical membranes and the role of the coiled-coil domain, will be interesting.
The manuscript uses "first-class" genetics to describe the different roles of the two Chitin synthases in Drosophila, comparing ectodermal chitin (tracheal and epidermal chitin) with endodermal (midgut) chitin. Such a precise analysis has not been investigated before in insects. Therefore, the study deeply extends knowledge about the role of Chitin synthases in insects. The audience will specialize in basic research in zoology, developmental biology, and cell biology regarding - how the different Chitin synthases produce chitin. Nevertheless, as chitin is relevant to material research and medical and immunological aspects, the manuscript will be fascinating beyond the specific field and thus for a broader audience.

I'm working on chitin in the tracheal system and epidermis in Drosophila.

Reviewer #2 (Evidence, reproducibility and clarity (Required)):

Drosophila have two different chitin synthase enzymes, Kkv and Chs2, and due to unique expression patterns and mutant phenotypes, it is relatively clear that they have different functions in producing either the cuticle-related chitin network (Kkv) or the chitin associated with the peritrophic matrix (PM). However, what is unknown is whether the different functions in making cuticle vs PM chitin is related to differences in cellular expression and/or enzyme properties within the cell. The authors exploit the genetic tractability of Drosophila and their ability to image cuticle vs PM chitin production to examine whether these 2 enzymes can substitute each other. They conclude that these two proteins are not equivalent in their capacity to generate chitin. The data are convincing; however, it is currently presented in a subjective fashion, which makes it difficult to interpret. Additionally, in my opinion there is some interpretation that requires softening or alternatively interpreted.

We are pleased that the reviewer finds our data convincing. However, we acknowledge the reviewer's concern that our data was presented in a subjective manner, and we apologise for this. In response, we have carefully reviewed the entire manuscript and revised our data presentation to ensure a more objective tone. Numerous changes (including additional quantifications, new experiments and clarifications) have been incorporated throughout the text. These revisions are highlighted in the marked-up version. We hope that this revision provides a more accurate and objective presentation of our work.

Major Comments:

1- While the imaging is lovely, there are some things that are difficult to see in the figures. For example, the "continuous, thin and faint 'chitin' layer that lined the gut epithelium" is very difficult to visualise in the control images. Can they increase the contrast to help the reader appreciate this layer? This is particularly important as we are asked to appreciate a loss of this layer in the absence of Chs2.

We have tried to improve the figures so that the PM layer in the midgut region is more clearly visible. We have added magnifications of small sections at the midgut lumen/epithelium border in grey to help visualise the PM. These improvements have been made in Figures 1,2,S1,S2,S3 and we believe that they better illustrate our results.

2- All the mutant analysis is presented subjectively. For example, the authors state that they "found a consistent difference of CBP staining when they compared the 'Chs2' escapers to the controls". How consistent is consistent? Can this be quantified? What is the penetrance of this phenotype? They say that the thin layer is absent in the midgut and the guts are thinner. Could they provide more concrete data?

As indicated above, we have reviewed the text to provide a more objective description of the phenotypes.
We have quantified the defects in the Df(Chs2) mutant conditions. For this quantification we dissected intestinal tracts of control and Df(Chs2) larva escapers. We fixed, stained and mounted them together. The control guts expressed GFP in the midgut region as a way to distinguish control from mutants. We analysed the presence or absence of chitin in the PM. We found absence of chitin in the proventricular lumen and in the midgut in all Df(Chs2) guts and presence of chitin there in all control ones (n=12 Df(Chs2) guts, n=9 control guts, from 5 independent experiments). The results indicate a fully penetrant phenotype of lack of chitin in Df(Chs2) larva escapers (100% penetrance). We have added this quantification in the text, chapter 2 (2. Chs2 deposits chitin in the PM).

To quantify the thickness of the guts, we took measurements of the diameter in control and Df(Chs2) guts at two comparable distance positions from the proventriculus (position 1, position 2, see image). Our quantifications indicated thinner tubes in mutant conditions.

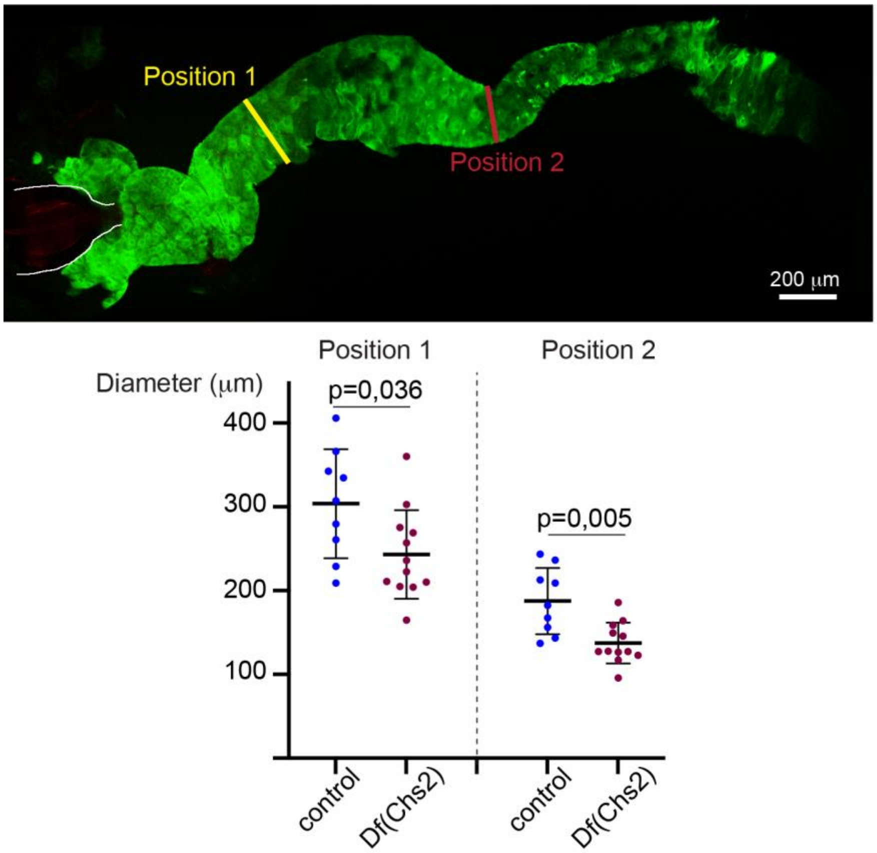

Image shows the anterior part of the intestinal tract, with the proventriculus encircled in white. Positions 1 and 2 indicate where the diameter quantifications were taken. Scatter plots quantifying the diameter at the two different positions in control and Chs2 larval escapers. Bars show mean ± SD. p=p value of unpaired t test two-tailed with Welch's correction.

However, we are aware that our analysis of the thickness of the gut is not accurate, because we have not used markers to precisely measure at the same position in all guts and because we have not normalised the measurement position in relation to the whole intestinal tract (mainly due to technical issues).

In relation to the fragility, we noticed that the guts of Chs2 larval escapers tended to break more easily during dissection than control guts, however, we have not been able to quantify this parameter in a reliable and objective manner.

Since we consider that the requirement of Chs2 for PM deposition is sufficiently demonstrated, and that aspects such as gut morphology or fragility relate to the physiological requirements of the PM, which we are beginning to address as a new independent project (see our response to point 5 of Reviewer 1), we have decided to remove the sentence 'We also noticed that the guts of L3 escapers were thinner and more fragile at dissection." from the manuscript to avoid subjectivity.

3- They state that Chs2 was able to restore accumulation of chitin in the PM of the proventriculus and the midgut. Please quantify. Additionally, does this restore the morphology of the guts (related to the comment above on the thinner guts in the absence of Chs2)?

We have quantified the rescue of chitin deposition in the PM when Chs2 is expressed in PR cells in a Df(Chs2) mutant background. For this quantification we used the following genetic cross: PRGal4/Cyo; Df(Chs2)/TM6dfdYFP (females) crossed to UASChs2GFP or UASChs2/Cyo; Df(Chs2)/TM6dfdYFP. We selected Df(Chs2) larval escapers by the absence of TM6 (recognisable by the body shape). Among these larval escapers, we identified the presence of Chs2 in PR cells by the expression of GFP or Chs2. We found absence of chitin in the proventriculus and in the midgut in all Df(Chs2) guts that did not express Chs2 in PR cells (n=8/8 Df(Chs2)). In contrast, chitin was present in those intestinal tracts where Chs2 expression was detected in PR cells (n=8/8 PRGal4-UASChs2; Df(Chs2) guts, from 5 independent experiments). The results indicate a full rescue of chitin deposition by Chs2 expression in PR cells in Df(Chs2) mutant larvae. We have added this quantification in the text, chapter 2 (2. Chs2 deposits chitin in the PM).

As requested by the reviewer, we have also conducted measurements to quantify gut thickness. We performed an analysis similar to the one described in point 2, this time comparing the diameter of Df(Chs2) and PRGal4-UASChs2;Df(Chs2) guts at positions 1 and 2 (see image in point 2 of Reviewer 2). Our quantifications indicated that guts were thicker when Chs2 is expressed in the PR region in Df(Chs2) larval escapers.

As discussed in point 2, we have decided not to include these results in the manuscript, as this type of analysis requires a more comprehensive investigation.

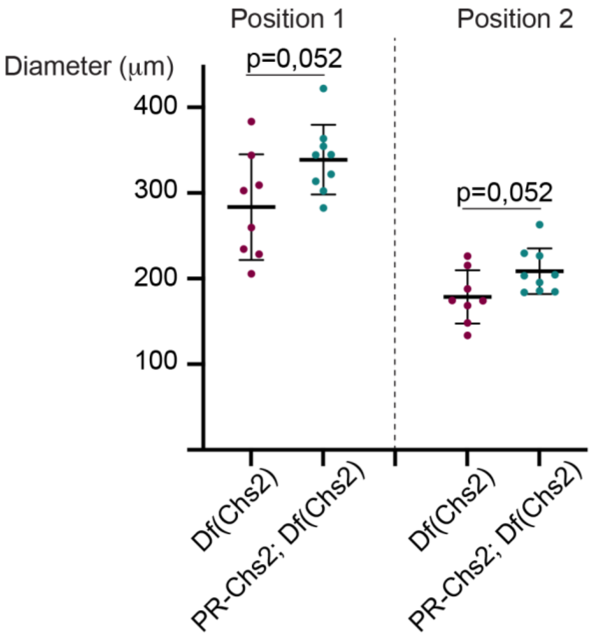

Scatter plots quantifying the diameter at the two different positions in Chs2 larval escapers and Chs2 larval escapers expressing Chs2 in PR cells. Bars show mean ± SD. p=p value of unpaired t test two-tailed with Welch's correction.

4- This may be beyond the scope of this paper, but I find it interesting that the PM chitin is deposited in the proventricular lumen. Yet it forms a thin layer that lines the entire midgut? Any idea how this presumably dense chitin network gets transported throughout the midgut to line the epithelium? I imagine that this is unlikely due to diffusion, especially if they see an even distribution across the midgut. Do they see any evidence of a graded lining (i.e. is it denser in the midgut towards the proventriculus and does this progressively decrease as you look through the midgut?)?

Insect peritrophic matrices have been classified into Type I and II (with some variations) depending on their origin (extensively reviewed in (Peters, 1992, Hegedus et al., 2019). Type I PMs are typically produced by delamination as concentric lamellae along the length of the midgut. Type II PMs, in contrast, are produced in a specialised region of the midgut that corresponds to the proventriculus and are typically more organised than Type I. In Type II PMs, distinct layers originate from distinct cell clusters in the proventriculus. It has been proposed that as food passes, it becomes encased by the extruded PM, which then slides down to ensheath the midgut. Drosophila larvae have been proposed to secrete a type II PM: through PM implantation experiments, Rizki proposed that the proventriculus is required to generate the PM in Drosophila larvae (Rizki, 1956). Our experiments confirmed this hypothesis: we show that expressing Chs2 exclusively in PR cells is sufficient to produce a PM along the midgut. Furthermore, we also show that expressing Chs2 in the midgut is not sufficient to produce a PM layer lining the midgut, at least at larval stages.

The type II PM in Drosophila is proposed to be fully organised into four layers in the proventricular region (also referred as PM formation zone) before reaching the midgut (Peters, 1992, King, 1988, Rizki, 1956, Zhu et al., 2024). However, the mechanism by which the PM is subsequently transported into the midgut remains unclear. PM movement posteriorly is thought to depend on to the pressure exerted by continuous secretion of PM material (Peters, 1992). Early work by Wigglesworth (1929, 1930) proposed that the PM is secreted into the proventricular lumen, becomes fully organised, and is then pushed down by a press mechanism involving the aposed ectodermal/endodermal walls of the proventriculus. Rizki suggested that muscular contractions of the proventriculus walls may play a role, and that peristaltic movements of the gut add a pulling force to push the PM into the midgut (Rizki, 1956). Nevertheless, to our knowledge, the exact mechanism is still not fully understood.

In response to the reviewer's question, the level of resolution of our analysis does not allow us to determine whether there is a graded PM lining along the midgut. However, available data using electron microscopy approaches suggest that the PM is a fully organised structure composed of four layers that is secreted and transported to line the midgut (King, 1988, Zhu et al., 2024).

5- The authors state that expression of kkv in tracheal cells of kkv mutants perfectly restores accumulation of chitin in the luminal filaments. Is this really 100% restoration? They also reference a paper here, which may have quantified this result.

We previously reported that the expression of kkv in tracheal cells restores chitin deposition in kkv mutants (Moussian et al,2015). However, our previous study did not quantify this rescue. As requested by the reviewer, we have now quantified the extent of the rescue.
To perform this quantification, we used the following genetic cross:

btlGa4/(Cyo); kkv/TM6dfdYFP (females) crossed to +/+; kkv UASkkvGFP/TM6dfdYFP (males)

We stained the resulting embryos with CBP (to detect chitin) and GFP. GFP staining allowed us to identify the kkv mutants (by the absence of dfdYFP marker) and to simultaneously identify the embryos that expressed kkvGFP in tracheal cells (through btlGal4-driven expression). Since btlGal4 is homozygous viable, most females carried two copies of btlGal4.
We compared the following embryo populations across 4 independent experiments:

1. Cyo/+; kkv/kkv UASkkvGFP (kkv mutants not expressing kkv in the trachea)
2. btlGal4/+; kkv/kkv UASkkvGFP (kkv mutants expressing kkv in the trachea)

Results:

1. Cyo/+; kkv/kkv UASkkvGFP ---- 0/6 embryos deposited chitin in trachea
2. btlGal4/+; kkv/kkv UASkkvGFP ---- 27/27 embryos deposited chitin in trachea

These results indicate complete restauration of chitin deposition in kkv mutants when kkv is expressed in tracheal cells (100% rescue).

To further investigate whether Chs2 can compensate for kkv function in ectodermal tissues, we performed a similar quantification using the following genetic cross:

btlGa4/(Cyo); kkv/TM6dfdYFP (females) crossed to UASChs2GFP/UASChs2GFP; kkv UASkkvGFP/TM6dfdYFP (males)

We compared the following embryo populations across 2 independent experiments:

1. Cyo/UASChs2GFP; kkv/kkv (kkv mutants not expressing Chs2 in the trachea)
2. btlGal4/ UASChs2GFP; kkv/kkv (kkv mutants expressing Chs2 in the trachea)

Results:

1. Cyo/UASChs2GFP; kkv/kkv ---- 0/4 embryos deposited chitin in trachea
2. btlGal4/ UASChs2GFP; kkv/kkv ---- 0/16 embryos deposited chitin in trachea

These results indicate no restauration of chitin deposition in kkv mutants expressing Chs2 in the trachea (0% rescue).

We have now incorporated these quantifications in the text, chapter 4 (4. Chs2 cannot replace Kkv and deposit chitin in ectodermal tissues.)

6- They ask whether Kkv overexpression in the proventriculus can rescue Chs2 mutants... and vice versa, whether Chs2 overexpression in ectodermal cells can rescue kkv mutants. They show that kkv overexpression leads to an intracellular accumulation of chitin in the proventriculus. However, Chs2 overexpression in the trachea did not lead to any accumulation of chitin in the cells. They tailored their experiments and the associated discussion to address the hypothesis that there is potentially some difference in trafficking of these components. However, another possibility, which they have not ruled out, is that the different ability of kkv and Chs2 to produce chitin inside cells of the proventriculus and ectoderm, respectively, is potentially related to different enzymatic activities and cofactors required for chitin formation in these different cell types. Is this another potential explanation for the differences that they observe?

We note that Kkv overexpression in any cell type (e.g. ectoderm, endoderm) consistently leads to chitin polymerisation. In ectodermal tissues, Kkv expression, in combination with Exp/Reb activity, results in extracellular chitin deposition. In the absence of Exp/Reb, Kkv expression leads to the accumulation of intracellular chitin punctae (De Giorgio et al., 2023, Moussian et al., 2015); this work). This correlates with the accumulation of Kkv at the apical membrane and presence of Kkv-containing vesicles, regardless of the presence of Exp/Reb (De Giorgio et al., 2023, Moussian et al., 2015); Figure 6, S6). In endodermal tissues, regardless of the presence of Exp/Reb, Kkv cannot deposit chitin extracellularly and instead produces intracellular chitin punctae. This correlates with a diffuse accumulation of Kkv in the endodermal cells (PR cells, or gut cells in the embryo) but presence of Kkv-containing vesicles (Figure 6, S6).

In previous work we showed that Kkv's ability to polymerise chitin is completely abolished when it is retained in the ER. Indeed, we found that a mutation in a conserved WGTRE region leads to ER retention, the absence of Kkv-containing vesicles in the cell, and absence of intracellular chitin punctae or chitin deposition (De Giorgio et al., 2023).

These findings indicate a correlation between Kkv subcellular localisation and chitin polymerisation/extrusion. Therefore, we hypothesise that intracellular trafficking and subsequent subcellular localisation play a crucial role in regulating Kkv activity (De Giorgio et al., 2023; this work).

We find that Chs2 is expressed in PR cells (Figure 4) and observe that only in these PR cells does Chs2 localise apically (Fig 5A-D, S5A,B). This localisation correlates with the ability of Chs2 to deposit chitin in the PM and the presence of intracellular chitin punctae in PR cells (Fig 1F). When Chs2 is expressed in other cells types, we detect it primarily in the ER and observed no Chs2-containing vesicles (vesicles are suggestive of trafficking). This localisation correlates with the inability of Chs2 to produce intracellular chitin punctae or extracellular chitin deposition.

Again, these results suggest a correlation between Chs2 subcellular localisation and chitin polymerisation/extrusion, aligning with the results observed for Kkv. Therefore, we hypothesise in this work that the intracellular trafficking and subsequent subcellular localisation of Chs2 play a crucial role in regulating its activity.

Our hypothesis is consistent with seminal work in yeast chitin synthases, which has demonstrated the critical role of intracellular trafficking, and particularly ER exit, in regulating chitin synthase activity (reviewed in (Sanchez and Roncero, 2022).

That said, we cannot exclude other explanations that are also compatible with the observed results. As pointed out by the reviewer, it is possible that Chs2 and Kkv require different enzymatic activities and/or cofactors for chitin polymerisation/deposition, which may be specific to different cell types. Indeed, we know that the auxiliary proteins Exp/Reb are specifically expressed in certain ectodermal tissues (Moussian et al., 2015). These mechanisms could act jointly or in parallel with the regulation of intracellular trafficking, or could even regulate this intracellular trafficking itself.
Identifying the exact mechanisms controlling Kkv and Chs2 intracellular trafficking would be necessary to determine whether additional mechanisms (specific cofactors or enzymatic activities) are also involved or even serve as the primary regulatory elements.
We have introduced these additional possibilities in the discussion section.

7- They co-express Chs2 and Reb and show that this does not lead to chitin production or secretion. In the discussion they conclude that Chs2 does not "seem to be dependent on 'Reb' activity". I think that this statement potentially needs softening. They show that Reb is not sufficient in to induce Chs2 chitin production in cells that do not normally make a PM. However, they do not show that it is not essential in cells that normally express Chs2 and make PM.

We fully agree with the reviewer's observation and thank her/him for pointing it out.
As indicated by the reviewer, we show that co-expression of Reb and Chs2 in different tissues does not lead to an effect distinct from that observed with Chs2 expression alone. In addition, in the discussion we mention that we could not detect expression of reb/exp in PR cells, which aligns with the findings from Zhu et al, 2024, indicating no expression of reb/exp in the midgut cells of the adult proventriculus, as assessed by scRNAseq. We found that exp is expressed in the ectodermal cells of the larval proventriculus (Fig S4D), correlating with kkv expression in this region and cuticle deposition. These findings led us to propose that Chs2 does not seem to be dependent on Exp/Reb activity.

However, in our original manuscript, we did not directly address whether Exp/Reb are required in the cells that normally express Chs2. As a result, we could not conclude that Chs2 relies on a set of auxiliary proteins different from Exp/Reb, and therefore a different molecular mechanism to that of Kkv in regulating chitin deposition.

To address this specific point, we have conducted a new experiment to test Exp/Reb requirement in PR cells. We co-expressed RNAi lines for Exp/Reb in these cells and found that chitin deposition in the PM was not prevented. This further supports the hypothesis that Exp/Reb activity is not necessary for Chs2 function. We have added this experiment to Chapter 4 and Fig S3I,J.

8- They looked at the endogenous expression pattern of kkv and Chs2 and say that they found accumulation of Kkv in the proventriculus and no accumulation in the midgut. Siimilarly, they look at the expression of Chs2 and detect it in cells of the proventriculus. Are there markers of these different cell types that they could use to colocalize these enzymes?

We agree with the reviewer that this is an important issue and we note that Reviewer 1 also raised the same point. Therefore, we have addressed this issue.

We obtained an antibody against Dve, kindly provided by Dr. Hideki Nakagoshi. Dve marks the endodermal region in the proventriculus (Fuss and Hoch, 1998, Fuss et al., 2004, Nakagoshi et al., 1998).This antibody worked nicely in our dissected L3 digestive tracts and allowed us to mark the endodermal region. We also obtained an antibody against Fkh, kindly provided by Dr. Pilar Carrera. Fkh marks the ectodermal foregut cells (Fuss and Hoch, 1998, Fuss et al., 2004, Nakagoshi et al., 1998). While, in our hands, this antibody performed well in embryonic tissues, we observed no staining in our dissected L3 digestive tracts. The reason for this is unclear, but we suspect technical limitations may be responsible (the ectodermal region of the proventriculus is very internal, potentially hindering antibody penetration). To circumvent this inconvenience, we tested a Fkh^{GFP} tagged allele available in Bloomington Stock Center. Fortunately, we were able to detect GFP in ectodermal cells of L3 carrying this allele. Using this approach, we conducted experiments to detect Fkh and Dve in relation to chitin accumulation in the wild type (Fig S1). In addition, we used these markers to map the expression of Kkv and Chs2 in the proventriculus (Fig 4). Our results using these endodermal/ectodermal markers confirmed the presence of a cuticle adjacent to the FkhGFP-positive cells and a PM adjacent to the PR cells, marked by Dve. Additionally, we show that Chs2-expressing cells are positive for Dve while Kkv-expressing cells are not. We could not conduct an experiment showing Kkv and Fkh co-expression due to technical incompatibilities, as we have to use GFP tagged alleles for both Kkv and Fkh to reveal their expression. However, we believe that our imaging of Dve/Kkv clearly shows that Kkv expressing cells lack Dve expression and localise in the internal (ectodermal) region of the proventriculus (Fig 4E).

9- They overexpress Chs2 in cells of the midgut and see that it colocalises with an ER marker. They conclude that it is retained in the ER, which again, for them suggests that it has a trafficking problem in these cells. However, they are overexpressing it in these cells and this strong accumulation that they observe in the ER could simply be due to the massive expression levels. Additionally, they cannot conclude that it doesn't get out of the ER at all. They could be correct in thinking that there may be a trafficking issue, but this experiment does not conclusively show that Chs2 is entirely retained in the ER when expressed in ectopic tissues. I wonder if their interpretation needs softening or whether they should potentially address alternative hypotheses.

The reviewer raises two distinct issues: 1) the localisation of overexpressed proteins 2) Chs2 ER retention.

We agree that massive overexpression can lead to artifactual subcellular localisation due to saturation of the secretory pathway, causing ER accumulation. In our experiments, we overexpressed Kkv and Chs2 in different tissues (trachea, salivary glands, embryonic gut, and larval proventriculus), inducing high levels of both chitin synthases.

For Kkv, we observed distinct subcellular localisation patterns in ectodermal versus endodermal tissues (illustrated in new Fig S6). In ectodermal tissues such as the trachea, large amounts of KkvGFP were detected, most of it localising apically. We also detected a more general KkvGFP distribution throughout the cell, including the ER, particularly at early stages. Additionally, we observed many KkvGFP-positive vesicles, reflecting exocytic and endocytic trafficking, as described previously (De Giorgio et al., 2023). The presence of these vesicles (as well as the apical localisation) indicates that KkvGFP is able to exit the ER. Indeed, our previous work demonstrated that when Kkv is retained in the ER, it does not localise apically or appear in vesicles (De Giorgio et al, 2023). In endodermal tissues, as described in our manuscript, KkvGFP did not exhibit polarised apical localisation and instead showed a diffuse pattern with some cortical enrichment. However, the presence of KkvGFP-containing vesicles still suggests that the protein is capable of exiting the ER also in these endodermal tissues.

We observed a different subcellular pattern when we overexpressed Chs2GFP. In tissues where Chs2 is not normally expressed (e.g., trachea, salivary gland, embryonic gut), we did not detect apical or membrane accumulation (see Fig. 5,S5, S6 and response to point 4 of Reviewer #1). Nor did we observe accumulation of Chs2GFP in intracellular vesicles. Instead, Chs2GFP showed strong colocalisation with an ER marker (see Fig. 5,S5, S6 and response to point 4 of Reviewer #1). In contrast, when overexpressed in PR cells, we detected apical enrichment (Fig 5A-D, S5A,B). This indicates that despite massive expression levels, Chs2 can exit the ER in particular tissues.

Taken together, our results strongly suggest that overexpressed Kkv can exit the ER in the different tissues analysed, whereas most Chs2GFP is retained in the ER in tissues other than PR cells. This correlates with the ability of overexpressed KkvGFP to polymerise chitin (either in intracellular puncta or deposited extracellularly depending on the presence of Exp/Reb) in all analysed tissues. Conversely, Chs2 was unable to polymerise chitin (either in intracellular puncta or extracellularly regardless of Exp/Reb presence) in tissues other than PR cells.

Nevertheless, we acknowledge that we cannot definitively conclude that all Chs2 protein is entirely retained in the ER. We have included this caveat in our revised manuscript (Chapter 6 and Discussion section).

Minor Comments:
- No mention of Fig 3I in the results section and the order discussed in the results does not match the order in the figure.

We apologise for these inconsistencies. We have addressed this issue in the text, figure legend, and the image order in Figure 3 and Figure S3.

- In the results please provide some information on what the CRIMIC collection is and how it allows you to see Chs2 expression for non-experts.

We have addressed this point in chapter 5 in the revised version, and we now provide a more detailed explanation of the CRIMIC Chs2$^{CR60212\text{-}TG4.0}$ allele.
Further details of this allele are also provided in our responses to points 5 and 9 of Reviewer 1.

Reviewer #2 (Significance (Required)):

Drosophila produce different types of chitinous structures that are required for either the exoskeleton of the animal or for proper gut function (peritrophic matrix). Additionally, most insects have two enzymes involved in the production of chitin and current data suggests that they have unique roles in producing either the exoskeleton or the peritrophic matrix. However, it is unclear whether their different functions are due to differences in cell type expression or differences in physiological activity of the enzymes. The authors exploit Drosophila to drive these 2 enzymes in different cell types that are known to produce the exoskeleton or the peritrophic matrix to determine whether they can functionally substitute mutant backgrounds. Their results give us a hint that these enzymes are not equivalent. What the authors were unable to address is why they are not equivalent. They hypothesise that the different physiological functions of the enzymes may be related to trafficking differences within their respective cell types. While this is an interesting hypothesis, the date are not really clear yet to make this conclusion.

This work will be of interest to anyone interested in chitinous structures in insects and the cell biology of chitin-related enzymes.

Literature

AHMAD, M., CHAUDHARY, S. U., AFZAL, A. J. & TARIQ, M. 2015. Starvation-Induced Dietary Behaviour in Drosophila melanogaster Larvae and Adults. *Sci Rep,* 5**,** 14285.

DE GIORGIO, E., GIANNIOS, P., ESPINAS, M. L. & LLIMARGAS, M. 2023. A dynamic interplay between chitin synthase and the proteins Expansion/Rebuf reveals that chitin polymerisation and translocation are uncoupled in Drosophila. *PLoS Biol,* 21**,** e3001978.

FUSS, B. & HOCH, M. 1998. Drosophila endoderm development requires a novel homeobox gene which is a target of Wingless and Dpp signalling. *Mech Dev,* 79**,** 83-97.

FUSS, B., JOSTEN, F., FEIX, M. & HOCH, M. 2004. Cell movements controlled by the Notch signalling cascade during foregut development in Drosophila. *Development,* 131**,** 1587-95.

HEGEDUS, D. D., TOPRAK, U. & ERLANDSON, M. 2019. Peritrophic matrix formation. *J Insect Physiol,* 117**,** 103898.

KING, D. G. 1988. Cellular organization and peritrophic membrane formation in the cardia (proventriculus) of Drosophila melanogaster. *J Morphol,* 196**,** 253-82.

LEBRETON, G. & CASANOVA, J. 2016. Ligand-binding and constitutive FGF receptors in single Drosophila tracheal cells: Implications for the role of FGF in collective migration. *Dev Dyn,* 245**,** 372-8.

MOUSSIAN, B., LETIZIA, A., MARTINEZ-CORRALES, G., ROTSTEIN, B., CASALI, A. & LLIMARGAS, M. 2015. Deciphering the genetic programme triggering timely and spatially-regulated chitin deposition. *PLoS Genet,* 11**,** e1004939.
NAKAGOSHI, H., HOSHI, M., NABESHIMA, Y. & MATSUZAKI, F. 1998. A novel homeobox gene mediates the Dpp signal to establish functional specificity within target cells. *Genes Dev,* 12**,** 2724-34.
PETERS, W. 1992. *Peritrophic Membranes*, Springer Berlin, Heidelberg.
RIZKI, M. T. M. 1956. The secretory activity of the proventriculus of Drosophila melanogaster. *Journal of Experimental Zoology,* 131**,** 203-221.
SANCHEZ, N. & RONCERO, C. 2022. Chitin Synthesis in Yeast: A Matter of Trafficking. *Int J Mol Sci,* 23.
ZHU, H., LUDINGTON, W. B. & SPRADLING, A. C. 2024. Cellular and molecular organization of the Drosophila foregut. *Proc Natl Acad Sci U S A,* 121**,** e2318760121.
