# Supplementary material for: Distinct cellular and molecular mechanisms contribute to the specificity of the two Drosophila melanogaster chitin synthases in chitin deposition
Source: PLoS Genet. 2025 Sep 8;21(9):e1011847. doi: 10.1371/journal.pgen.1011847 (PMC12431666; doi:10.1371/journal.pgen.1011847)
Supplement: Attachment — Submitted filename: Editor and Reviewers responses.pdf [file pgen.1011847.s011.pdf]

Additional Editor Comments:

I would like you to address the minor criticisms of reviewers' 2 & 4, which do not require additional experiments but are intended to benefit the readership. But you can ignore reviewer 2's comments about deleting the chimeras.

We thank the reviewers for the positive comments.
We have addressed the criticisms of Reviewers 2 and 4 in the manuscript text and Figure 1. The changes made to the manuscript are highlighted in blue in the marked-up revised version.

Reviewer #2:

Previous reviews of this manuscript were detailed and comprehensive. I fully agree with both reviewers' assertions that the work is important and beautifully executed. For the most part, the authors addressed the reviewers' main points nicely in this revised version (but see below). I will be interested to see what their work on physiological mechanisms reveals in future submissions. I do wonder if in their experiment #2 to address Reviewer #1's point 3, where they starved larvae, they were able to detect a change in the deposition of chitin in the midgut (Reviewer #2's point 4). If one of the ideas for how the PM is distributed is from food pushing through the gut, one might expect it to be less organized in the starved larvae.

To satisfy the reviewer's curiosity, we can report that we have detected PM deposition in both the proventriculus and midgut under the various starvation conditions tested. These findings would suggest that, in *Drosophila*, PM deposition is not directly linked to food intake. We are currently continuing these experiments to gain a deeper understanding of the process.

The authors may have misunderstood Reviewer 2's points 3 and 5, which asks them to quantify the restoration of chitin in mutants. I suspect that the reviewer was asking if the amount of chitin was fully restored, not how many animals were examined and showed detectable deposition of chitin. In their rebuttal, the authors use terms like "full rescue" and "100% restoration" when they just mean that chitin was detected, not that the amount detected is equivalent to the wild-type level. While documenting the penetrance is helpful, they should not use absolute terms that imply quantification of amount when they are simply measuring presence vs. absence of chitin-binding protein fluorescence. I note that on page 7 the authors state "Chs2 expression was able to restore the chitin accumulation in the PM" which implies full restoration. This could be softened to "was able to restore detectable chitin accumulation". On page 9, they state "expression of kkv in tracheal cells of kkv mutants fully restores the accumulation of chitin in the luminal filament and in the tracheal cuticle (100% rescue, n=27/27 …". Again, the term "fully restores" is not appropriate if the absolute amount of chitin is not measured. Finally, in the revised discussion they state "expression of Chs2 exclusively in the PR cells is sufficient to

produce all the chitin deposited in the PM", where saying "all" may not be accurate since the amount was not quantified.

The reviewer is correct in this observation. In response to the original comments from Reviewer 2, we quantified the penetrance of the rescue/no rescue phenotypes. However, we did not quantify the amount of chitin produced under the different conditions, as the immunofluorescence images lack the resolution required for reliable quantitative analysis. As suggested by the reviewer, we have addressed this point in the text. The changes made to the manuscript are highlighted in blue in the marked-up revised version.

My only remaining question about this manuscript is whether the experiments analyzing the chimeric molecules should be included. Neither chimeric construct is correctly localized, and both appear to be retained in the ER. This suggests that the novel molecules fail to fold properly or have some internal incompatibility that causes aggregation. The negative results presented in Fig. 7 do not seem to contribute anything meaningful to this paper. Perhaps in future a more surgical approach – inserting the coiled-coil domain alone into the Chs2 sequence or swapping the multi-TM-spanning domains in the amino-termini – might be more informative.

We understand the reviewer's point, however, we believe that the experiments involving the chimeric constructs provide valuable information. We found that both protein chimeras localise in the ER. Because we previously showed that the absence of the coiled coil domain in Kkv does not prevent the protein to exit the ER, reach the apical membrane and to be active (De Giorgio et al., 2023), we speculate that the two chimeras are unable to properly fold or oligomerise in the ER, which may impair their ability to reach the membrane. Oligomerisation is known to be a critical step in yeast CHS and is required for proper localisation and function. Our findings may suggest that Drosophila CHS trafficking and activity require oligomerisation involving both the N-terminal and C-terminal domains.

Overall, I found the data nicely support the authors' conclusions and the paper represents a solid advance in our understanding of chitin deposition in insects.

Minor comments:
In fig. 1B, it might be helpful to make a dotted line box outlining the portion of the proventriculus being represented in fig. 1C.

We have addressed this point in the corrected Figure 1

Typos:
Pg. 4 "in a specific a region of the proventriculus"
Pg 7 "penentrant"
Pg. 18 "glycosilated"
Pg. 31 "shematic" (twice)

We have corrected the typos

Reviewer #4:

The manuscript was submitted along with two full reviews communicated by Review Commons. These reviews cover the subject in detail and served to improve the presentation of the work considerably. Besides the points raised by the reviewers, I would like to propose the following three points to be addressed in addition:
1) The introduction does not give enough information on the data and concept of chitin synthesis machinery available for insects, where several proteins mentioned in the results and discussion section are known to assist the central enzyme chitin synthase (mainly the epidermal one: SERCA, Reb, Knk/Rtv etc). This information should be given in the introduction and point by point discussed in the discussion.

In the original submission, we introduced the roles of Exp/Reb in the Introduction, evaluated their involvement in PM deposition in the Results section, and discussed their function in the Discussion. In this revised version we have added information about other identified auxiliary proteins in insects, both in the Introduction and briefly in the Discussion. These additions are highlighted in blue in the marked-up revised version. As our study did not investigate the role of auxiliary proteins beyond Exp/Reb, we have kept this section concise to maintain focus and avoid excessive length.

2) In the same direction, the concept of chitosomes in fungi/yeasts should/could be introduced and discussed as comparison with the fungal system has already been included in the manuscript.

We have introduced the concept of chitosomes in the Discussion. This addition is highlighted in blue in the marked-up revised version.

3) Ther type 2 peritrophic matrix (PM) has been proposed to issue from chitin synthase activity in the PR cells only. To follow the argument, a more detailed description of PM types should be given.

We have revised the Introduction and Discussion to include a more detailed yet concise description of the different types of PM. The changes are highlighted in blue in the marked-up revised manuscript.
